# Home-Based Rehabilitation of the Shoulder Using Auxiliary Systems and Artificial Intelligence: An Overview

**DOI:** 10.3390/s23167100

**Published:** 2023-08-11

**Authors:** Bruno Cunha, Ricardo Ferreira, Andreia S. P. Sousa

**Affiliations:** 1 Center for Rehabilitation Research—Human Movement System (Re)habilitation Area, Department of Physiotherapy, School of Health-Polytechnic of Porto, Rua Dr. António Bernardino de Almeida, 400, 4200-072 Porto, Portugal; andreia.asps@gmail.com; 2 Institute for Systems and Computer Engineering, Technology and Science—Telecommunications and Multimedia Centre, FEUP, University of Porto, Rua Dr. Roberto Frias, 4200-465 Porto, Portugal; ricardomsf1999@outlook.pt

**Keywords:** home-based rehabilitation, wearables, robots, exoskeletons, machine learning, virtual reality, augmented reality, serious games

## Abstract

Advancements in modern medicine have bolstered the usage of home-based rehabilitation services for patients, particularly those recovering from diseases or conditions that necessitate a structured rehabilitation process. Understanding the technological factors that can influence the efficacy of home-based rehabilitation is crucial for optimizing patient outcomes. As technologies continue to evolve rapidly, it is imperative to document the current state of the art and elucidate the key features of the hardware and software employed in these rehabilitation systems. This narrative review aims to provide a summary of the modern technological trends and advancements in home-based shoulder rehabilitation scenarios. It specifically focuses on wearable devices, robots, exoskeletons, machine learning, virtual and augmented reality, and serious games. Through an in-depth analysis of existing literature and research, this review presents the state of the art in home-based rehabilitation systems, highlighting their strengths and limitations. Furthermore, this review proposes hypotheses and potential directions for future upgrades and enhancements in these technologies. By exploring the integration of these technologies into home-based rehabilitation, this review aims to shed light on the current landscape and offer insights into the future possibilities for improving patient outcomes and optimizing the effectiveness of home-based rehabilitation programs.

## 1. Introduction

The shoulder joint, renowned for its exceptional range of movement, plays a crucial role in enabling essential daily activities, such as reaching, lifting, or throwing [1]. Coordinated movement between the humerus, scapula, clavicle, thoracic wall, and thoracic spine is vital for optimal shoulder function, and injuries to these structures can significantly impair shoulder motion [2]. With advancing age, the incidence of common shoulder pathologies, including tendinopathy and osteoarthritis, rises progressively, affecting a substantial portion of individuals aged 65 and above [3,4,5]. Notably, rotator cuff tears pose a significant concern, afflicting approximately one-fifth of the global adult population and up to half of those aged 66 years and above [6]. The prevalence of upper limb pain among adults is striking, with 36% of the population reporting such pain and a substantial portion of these cases contributing to significant morbidity, healthcare resource consumption, and work productivity loss. Hence, the proper evaluation and treatment of shoulder pathologies assume paramount importance [7].

Recognized as an alternative or complementary approach to traditional care-unit-based therapy, home-based rehabilitation empowers patients by fostering autonomy and facilitating their recovery to regain their ability to perform daily tasks [8]. This approach offers numerous advantages for patients and healthcare organizations, including resource optimization, reduced travel time, flexible appointment scheduling, improved therapist availability, and prompt feedback delivery [9]. Consequently, home-based rehabilitation enables the transition of patients who would otherwise require in-patient care to an outpatient setting, where they actively engage in prescribed rehabilitation exercises at home, promoting adherence to the treatment regimen [10,11,12].

Maintaining high-quality healthcare services in a home-based rehabilitation setting necessitates the use of medical devices capable of monitoring patients, complemented with periodic supervisions from healthcare professionals [13]. Well-designed medical devices should ensure reliable clinical treatment, prioritize patient safety and well-being, and safeguard the caregivers and healthcare providers involved [14]. In recent years, advancements in remote monitoring technologies, particularly in e-health, have facilitated the migration of various devices from hospital settings to home-based paradigms. These devices, often managed by non-health professionals, are now designed to be more compact and smaller, ergonomic, and user friendly, catering to the needs of diverse users [15].

While previous literature reviews have explored specific technological factors, applicable exercises, and their effectiveness in home-based shoulder rehabilitation [16,17,18,19], this narrative review aims to provide a comprehensive understanding of state-of-the-art technologies in this field, distinguishing it from existing literature work, which has not shared this specific objective. By adopting a beginner-friendly approach, this review offers researchers starting their careers in the rehabilitation field a broad perspective and a solid foundation to explore the various technological advancements associated with home-based shoulder rehabilitation.

The present article is organized into five sections, each addressing specific aspects of home-based shoulder rehabilitation. Section 1 introduces the objectives and themes of this review, setting the foundation for the subsequent sections. In Section 2, a comprehensive overview of auxiliary technologies employed in home-based shoulder rehabilitation is presented, showcasing concrete examples from both the physical and virtual domains. Section 3 delves into the exploration of therapeutic exercises commonly utilized in shoulder rehabilitation and assesses their suitability for home-based environments. Section 4 puts forward a set of guidelines to ensure optimal user experience, safety, and positive rehabilitation outcomes on home-based rehabilitation systems. Section 5 discusses the limitations encountered during the writing process of this article, while also acknowledging potential areas for future research. Finally, Section 5 summarizes the key findings and conclusions.

## 2. Technology Solutions for Home-Based Shoulder Rehabilitation

**Figure 1:**
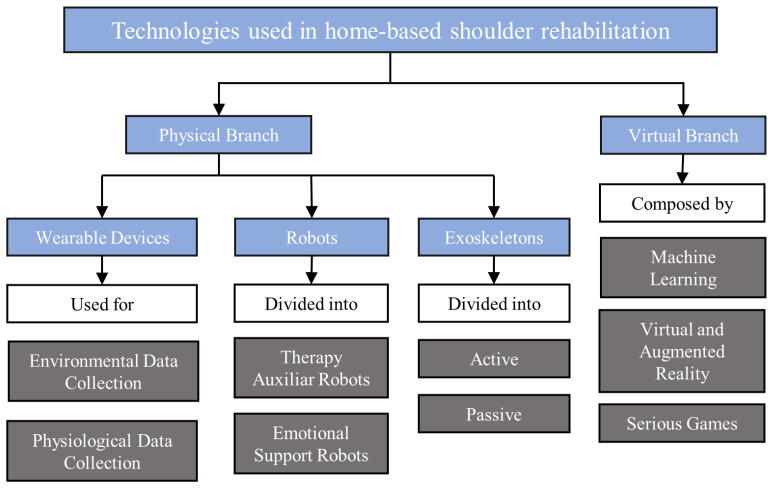
Technologies used for home-based shoulder rehabilitation.

In the realm of medical advancements, numerous devices are currently under development, with many designed for use in clinical or hospital settings due to their inherent complexity. However, certain devices and technologies offer relative simplicity in their operation, making them well suited for home use and enabling patients to regain autonomy and independence in their rehabilitation journey [20]. Figure 1 presents an overview of these key technologies, categorized into the domains of physical and virtual applications, each playing a significant role in home-based rehabilitation. By exploring these technologies, we can better understand their potential impact and effectiveness in supporting patients’ rehabilitation progress.

Within the realm of physical technologies, wearable devices, also known as wearables, have emerged as a transformative tool in modern medicine. These devices incorporate sensors, which gather data from the patient and/or the surrounding environment, facilitating continuous monitoring outside traditional healthcare settings. Through the utilization of advanced computational algorithms, wearables enable event prediction, prevention, and intervention, thus revolutionizing the field [21]. Notably, wearable devices offer a promising avenue for long-term monitoring and follow-up in the context of home-based shoulder rehabilitation, as evidenced by recent scientific publications [22,23,24,25,26]. By harnessing the potential of wearables, healthcare providers can gather valuable insights and optimize the rehabilitation process to enhance patient outcomes and recovery.

The integration of rehabilitation robots has been a pivotal aspect of therapy since the 1990s, serving as a valuable aid in administering treatment to patients. These robots employ computerized interfaces to assist individuals in completing tasks, offering not only physical support but also emotional encouragement and motivation. Numerous studies have demonstrated the positive impact of robot-assisted training on arm function and overall ability in daily activities [27,28,29,30]. Additionally, exoskeletons, which are wearable robotic units designed to augment and enhance human physical capabilities, have gained prominence, particularly among individuals with reduced mobility [31]. The growing demand for upper limb exoskeletons has led to the development of new and advanced models in recent times [32,33,34,35]. By harnessing the potential of rehabilitation robots and exoskeletons, patients can benefit from enhanced therapy outcomes and improved quality of life.

In the realm of virtual approaches to home-based rehabilitation, the integration of machine learning has revolutionized the automation of processes by harnessing artificial intelligence and pattern recognition capabilities [36]. Through the analysis of empirical data collected from sensors embedded in modern smartphones and wearables, machine-learning algorithms can gather user and environmental information, enabling real-time adaptation to the prevailing conditions, as if the systems were continuously learning and evolving [36]. Deep learning, a subset of machine learning, has particularly contributed to remarkable advancements in image recognition and behavior prediction within the field of rehabilitation [37]. Over the years, deep learning has demonstrated its efficacy in shoulder rehabilitation, exemplified by numerous studies, which will be explored in this article [36,38,39,40]. By leveraging the power of machine learning and its subfield of deep learning, home-based rehabilitation can capitalize on sophisticated algorithms to optimize treatment outcomes and refine personalized interventions.

The increasing adoption of virtual and augmented reality systems in recent years indicates a shift toward a more e-health-focused approach in both medical and social rehabilitation [41]. These systems have the capability to simulate realistic scenes, providing valuable support to individuals with disabilities throughout the rehabilitation process. Notably, the literature showcases various examples where virtual and augmented reality technologies have been successfully employed to enhance rehabilitation outcomes [42,43,44,45,46]. By immersing users in interactive and lifelike environments, these technologies offer new avenues for therapeutic interventions and enable individuals to engage in meaningful and immersive rehabilitation experiences.

A compelling concept, which has emerged in the realm of physical rehabilitation, is the use of serious games. These games combine educational, informational, and rehabilitative aspects with the entertaining and enjoyable elements of video games [47]. The versatility of gamification in rehabilitation is vast, as it can leverage various technologies, such as virtual reality or robots [48,49,50,51]. By integrating game mechanics, challenges, and rewards, serious games provide an engaging and motivating platform for individuals undergoing rehabilitation, encouraging active participation and facilitating progress tracking. The integration of serious games into home-based rehabilitation programs has the potential to transform therapy into a more enjoyable and interactive experience, ultimately improving patient engagement and treatment outcomes.

With the foundational technologies discussed in this section, the subsequent sections will delve deeper into their key features, providing illustrative examples and conducting in-depth analyses. By exploring the capabilities and applications of these technologies, we aim to offer valuable insights into the evolving landscape of home-based shoulder rehabilitation and equip researchers and practitioners with the knowledge needed to harness these advancements effectively.

### 2.1. Physical Branch

The physical branch of rehabilitation encompasses a range of technologies, which integrate robotics to enhance the treatment approaches for muscular, neuromuscular, and osseous conditions [52]. This branch primarily focuses on the development and utilization of mechanical devices designed to monitor and interact with patients, offering support and assistance throughout the rehabilitation journey [53]. Wearable devices, robots, and exoskeletons form the core components of this branch, as illustrated in Figure 1, facilitating and augmenting the rehabilitation process by providing personalized interventions and real-time feedback.

#### 2.1.1. Wearable Devices

Wearable sensors play a crucial role in shoulder rehabilitation, particularly for individuals with neurological or musculoskeletal impairments. These sensors enable the assessment of motor abilities by extracting and analyzing kinematics data, facilitating rehabilitation follow-up and treatment of disorders [54]. Table 1 provides examples of wearable devices used in home-based shoulder rehabilitation. Several wearable devices have been developed for this purpose, such as an affordable upper body orthotics system with cable actuation, a soft-orthotic system for posture monitoring and control, a motion sensor device for assisting home-based exercises, a portable system without additional sensors or markers, and a lightweight soft-orthotic device with integrated cable actuation and limb position sensing. These devices aim to provide support, feedback, and assistance during shoulder rehabilitation exercises, offering potential solutions to enhance home-based rehabilitation programs and improve patient outcomes.

The outcomes of wearable applications in home-based shoulder rehabilitation have been predominantly positive, ensuring patient safety and yielding rehabilitation results comparable to those observed in a care unit [51,55]. For instance, Condino et al. [43] successfully tested these approaches with a group of five rehabilitation specialists and twenty healthy subjects, demonstrating the ergonomic design and motivational value of these interventions, which added positive value to the rehabilitation process.

#### 2.1.2. Robots

Over the past two decades, significant advancements in the fields of robotics and orthotics have positively impacted the telerehabilitation process and motor function recovery outcomes in physical therapy. These technical improvements have led to enhanced patient experiences and better therapeutic results [56]. Some examples of these robots are presented in Table 2.

The integration of robotic and orthotic technologies has brought about several key benefits. Robotic devices can provide real-time feedback, enabling patients to receive immediate information about their movements and posture. This feedback enhances patients’ understanding and correction of their movements, leading to improved motor function recovery. Moreover, the feedback from robots can boost patient motivation and adherence to rehabilitation therapy, as it provides a sense of accomplishment and progress. Robotic devices also offer physical assistance, supporting patients in performing exercises and movements. By augmenting patients’ abilities, robots enable more intensive and targeted therapy, potentially leading to better motor function recovery outcomes. Furthermore, the combination of telecommunication technologies with robotics has facilitated telerehabilitation. Telerehabilitation allows patients to access therapy remotely, reducing the need for in-person visits. Patients can engage in rehabilitation exercises and receive guidance from healthcare professionals through video conferencing or other communication tools, while robotic devices aid them in performing the prescribed activities.

While the use of robotic devices autonomously in home rehabilitation settings is possible, safety measures and guidelines must be followed. Robotic devices designed for home use incorporate safety features to prevent excessive force or unexpected movements. However, the level of supervision required may vary depending on the patient’s condition and the complexity of the device. Initial setup and training by healthcare professionals may be necessary, and regular check-ins or remote monitoring can ensure progress tracking and program adjustments [60].

The technical improvements in the robotic and orthotic fields have significantly improved the telerehabilitation process and motor function recovery outcomes in physical therapy. These advancements have allowed patients to receive real-time feedback, physical assistance, and guidance from healthcare professionals remotely. While patients can use robotic devices autonomously in their homes, the level of supervision required depends on individual factors and should be determined in consultation with healthcare professionals to ensure safety and optimal outcomes.

#### 2.1.3. Exoskeletons

Exoskeletons are described as wearable robotic devices, designed with the intent of facilitating and enhancing the user’s physical activities in a safe way, normally applied in patients with reduced mobility, such as patients who suffered spinal cord injuries [61] or strokes [62]. It is possible to divide exoskeletons into two big groups, namely active and passive exoskeletons, where active exoskeletons contain one or more actuators, which actively augment the physical capabilities of the user using electrical motors, while passive exoskeletons resort to materials such as springs and dampers to store the energy produced during human movement and then release it to help during the subsequent movements [63]. Even though a distinction between active and passive exoskeletons exists, there can also be exoskeletons, which incorporate both approaches [64]. They can also present different degrees of freedom (DOF), ranging from as low as 3 DOF [65] or having 10 DOF or more [66]. Some examples of exoskeletons are detailed in Table 3.

When applied in upper limb rehabilitation, exoskeletons are primarily used to sustain a proper and ergonomic posture and give a boost of strength to the shoulder [68]. In order to do so, exoskeletons need to have some data inputs, with some of the most common inputs being the patient’s electromyography (EMG) signal [69], encephalography (EEG) signal [70], and force of torque [71], all acquired throw sensors embedded in the exoskeleton.

### 2.2. Virtual Branch

The virtual branch of rehabilitation encompasses a wide array of informatics technologies, such as machine learning and its subfields, virtual and augmented reality, and serious games. These technologies are pivotal in simulating human cognitive capabilities and enabling the control of health management systems, thereby automating decision-making processes in clinical support systems [53]. By harnessing the power of these informatics tools, the virtual branch of rehabilitation significantly contributes to the advancement of rehabilitation practices.

One of the key aspects of the virtual branch is the creation of immersive and interactive experiences. Through virtual and augmented reality technologies, patients can engage in rehabilitation activities within simulated environments, which closely resemble real-world scenarios. This immersive approach provides a controlled and safe space for patients to practice various tasks, such as balance training, motor skill development, and functional movements. By replicating real-life situations, virtual environments allow patients to work on their rehabilitation goals in a dynamic and engaging manner. Furthermore, the virtual branch of rehabilitation offers personalized interventions, which cater to individual patient needs. Machine-learning techniques are employed to analyze patient data, predict outcomes, and optimize treatment plans. By leveraging personalized data, rehabilitation programs can be tailored to specific patient characteristics, resulting in more targeted and effective interventions. The utilization of machine learning and its subfields empowers healthcare professionals to make data-driven decisions and adapt rehabilitation strategies based on individual progress and requirements.

Intelligent data analysis is another significant aspect of the virtual branch. By employing advanced analytics and machine-learning algorithms, large datasets collected from patients can be processed and analyzed to derive meaningful insights. This data-driven approach aids in identifying patterns, trends, and correlations, which can inform clinical decision making, treatment protocols, and the development of evidence-based rehabilitation practices. The ability to extract valuable knowledge from vast amounts of data enhances the understanding of patient outcomes, therapy effectiveness, and the overall impact of rehabilitation interventions.

The virtual branch of rehabilitation integrates informatics technologies, such as machine learning, virtual and augmented reality, and serious games, to revolutionize rehabilitation practices (these are further expanded on in the following subsections). Through immersive and interactive experiences, personalized interventions, and intelligent data analysis, this branch offers new possibilities for enhancing patient outcomes, advancing clinical decision making, and promoting evidence-based rehabilitation strategies.

#### 2.2.1. Machine Learning

The incorporation of machine-learning algorithms in rehabilitation has gained significant attention over the past two decades. Much of the research in this field has focused on utilizing machine-learning algorithms for classification, prediction, and treatment planning [72,73]. The aim is to develop intelligent rehabilitation systems capable of automatically adapting to patients’ needs and providing tailored interventions based on individual features [74]. Several noteworthy examples of machine-learning applications in rehabilitation are presented in Table 4, such as the iJoint [38], a telerehabilitation system, which uses a smartphone to estimate the angle of rotation, showcasing the diverse range of techniques employed. 

The adoption of rehabilitation systems integrating machine-learning techniques has further accelerated in the wake of the global COVID-19 outbreak. These systems offer several advantages, including remote access to patients’ exercise information without the need for direct physical contact, enabling health professionals to monitor their patients’ progress at any time [75]. This remote supervision capability not only ensures continuous care but also allows health professionals to simultaneously oversee the progress of multiple patients, significantly enhancing their productivity. By leveraging machine learning and remote monitoring, rehabilitation systems are transforming the way healthcare professionals deliver care, particularly in times of unprecedented challenges.

#### 2.2.2. Virtual and Augmented Reality

Since the early 1990s, there has been a growing development of physical rehabilitation interventions utilizing virtual reality (VR) technology, with laboratories and clinics promoting its use in healthcare [76]. VR provides an immersive environment, which allows individuals to interact with computer-generated stimuli, while augmented reality (AR) systems merge virtual objects with real-world objects, enabling user interaction with virtual elements [77]. Table 5 presents examples of VR and AR applications applied to home-based shoulder rehabilitation.

The use of VR and AR applications in the recovery of cognitive function and simulation of daily life activity has shown great promise in the field of home-based rehabilitation [78]. These technologies offer several advantages, including the creation of a controllable environment, inducing a sense of presence, providing entertaining treatments, and generating digital records of rehabilitation sessions. Furthermore, the incorporation of mental practice as an additional tool for motor rehabilitation is gaining importance in newer treatments, which utilize artificial motor imagery to enhance motor behavior. Visual representations of specific motor actions are employed to engage the patient’s working memory, and VR and AR systems play a crucial role in facilitating these approaches [79]. By leveraging VR and AR technologies, rehabilitation programs can offer innovative and effective methods for enhancing motor recovery and promoting engagement in the rehabilitation process.

#### 2.2.3. Serious Games

Serious games have gained considerable attention from researchers as a promising component in clinical practice—particularly for the rehabilitation of motor dysfunctions—over the past three decades [80]. These games can be implemented in various systems, and popular gaming consoles have also embraced this technology, often incorporating VR to enhance the gaming experience. For instance, Microsoft Kinect^®^ has been utilized by Xbox [81,82]; Nintendo Wii^®^ has offered a distinctive remote controller and accessories [82,83]; and PlayStation Move^®^ has been developed by PlayStation [82]. Kinect^®^ [81,82] uses a motion-sensing camera to track the user’s movements and gestures, allowing them to interact with virtual environments and games on a screen. The advantages of this system are that it does not require any wearable devices or markers; it is relatively affordable and widely available; and it offers a variety of games and exercises for different levels of difficulty and goals. The disadvantages are that it may not be very accurate or reliable in measuring the user’s movements; it may not provide sufficient feedback or guidance to the user; and it may not be suitable for users with severe impairments or limited mobility. Nintendo Wii^®^ [82,83] uses a wireless remote controller and accessories to detect the user’s movements and gestures, allowing them to interact with virtual environments and games on a screen. The advantages of this system are that it is easy to use and set up; it is relatively affordable and widely available; and it offers a variety of games and exercises for different levels of difficulty and goals. The disadvantages are that it may not be very accurate or reliable in measuring the user’s movements; it may not provide sufficient feedback or guidance to the user; and it may cause fatigue or discomfort due to the weight or shape of the controller or accessories. PlayStation Move^®^ [82] uses a wireless motion controller and a camera to track the user’s movements and gestures, allowing them to interact with virtual environments and games on a screen. The advantages of this system are that it is the most accurate and reliable in measuring the user’s movements; it provides more feedback and guidance to the user; and it offers a variety of games and exercises for different levels of difficulty and goals. The disadvantages are that it is more expensive and less available; it requires more space and equipment to set up; and it may cause fatigue or discomfort due to the weight or shape of the controller. Table 6 provides additional examples of serious gaming applications in shoulder rehabilitation.

The development of serious games has focused on tailoring the gaming environment to specific disease conditions, aiming to motivate patients to complete exercises and improve clinical outcomes [84]. By creating an engaging and interactive experience, serious games provide a motivating platform, which encourages patients to actively participate in their rehabilitation. The incorporation of game elements, such as challenges, rewards, and progression, enhances patient engagement and increases adherence to the prescribed exercises. As a result, serious games have the potential to boost the effectiveness of shoulder rehabilitation programs and contribute to improved clinical outcomes.

#### 2.2.4. Methodologies and Measuring Parameters in Existing Literature

In the existing literature, a variety of methodologies and measuring parameters have been used to evaluate the effectiveness of auxiliary technologies in home-based shoulder rehabilitation [85,86]. These include randomized controlled trials, case studies, and observational studies, which are detailed below.

Randomized controlled trials are considered the gold standard for evaluating the effectiveness of medical interventions [15]. In these studies, participants are randomly assigned to either a treatment group (receiving the auxiliary technology) or a control group (receiving standard care). The outcomes of the two groups are then compared to determine the effectiveness of the intervention. This type of study design helps to control for potential confounding factors and provides strong evidence for the effectiveness of an intervention.

Case studies and observational studies, on the other hand, involve observing and documenting the experiences of individual patients or groups of patients using auxiliary technologies [55,87]. These studies can provide valuable insights into the real-world use and effectiveness of these technologies. However, they are often limited by their lack of control over potential confounding factors and may not provide strong evidence for the effectiveness of an intervention.

In terms of measuring parameters, a variety of metrics have been used to evaluate the performance of auxiliary technologies in home-based shoulder rehabilitation [88]. These include range of motion, strength, pain, and functional outcomes, such as activities of daily living. Range of motion is typically measured using goniometers or inclinometers, while strength is measured using dynamometers or manual muscle testing. Pain is often assessed using visual analog scales or numerical rating scales, while functional outcomes are assessed using standardized questionnaires or performance-based tests.

In addition to these objective measures, patient-reported outcome measures, such as quality of life and satisfaction with treatment, have also been used [88,89]. These measures provide valuable information about the patient’s subjective experience and can help in assessing the overall impact of an intervention on their well-being.

From our experience, we believe that a comparison of the results obtained using different methodologies usually reveals some interesting trends and patterns. For example, randomized controlled trials tend to produce more consistent and reliable results than case studies or observational studies. However, these studies can be time consuming and expensive to conduct, and they may not always reflect real-world use of auxiliary technologies.

In conclusion, home-based rehabilitation employs a variety of methodologies and measuring parameters. While randomized controlled trials are considered the gold standard for evaluating medical interventions, case studies and observational studies can also provide valuable insights. A range of metrics have been used to measure the performance of these technologies, including range of motion, strength, pain, and functional outcomes.

#### 2.2.5. Summary

Physical methods include the use of wearable devices, robots, and exoskeletons. Wearable devices incorporate sensors, which gather data from the patient and/or the surrounding environment, facilitating continuous monitoring outside traditional healthcare settings. Robots employ computerized interfaces to assist individuals in completing tasks, offering not only physical support but also emotional encouragement and motivation. Exoskeletons are wearable robotic units designed to augment and enhance human physical capabilities.

Virtual methods include the use of machine learning, virtual and augmented reality, and serious games. Machine-learning techniques are employed to analyze patient data, predict outcomes, and optimize treatment plans. Virtual and augmented reality systems simulate realistic scenes, providing valuable support to individuals with disabilities throughout the rehabilitation process. Serious games combine educational, informational, and rehabilitative aspects with the entertaining and enjoyable elements of video games.

Each method has its own advantages and disadvantages. Physical methods offer real-time feedback, physical assistance, and remote monitoring capabilities. However, they may require initial setup and training by healthcare professionals, and regular check-ins or remote monitoring may be necessary to ensure progress tracking and program adjustments. Virtual methods offer immersive and interactive experiences, personalized interventions, and intelligent data analysis. However, they may require specialized equipment or software, and their effectiveness may vary depending on individual factors, such as cognitive function or motivation.

## 3. Considerations for Therapeutic Exercises in Home-Based Rehabilitation

Enhancing shoulder functionality through physical rehabilitation is a crucial aspect of achieving effective recovery following injury or surgery. This process entails engaging in appropriate rehabilitation exercises, which target various movement planes to enhance motor function [90]. By providing targeted stimuli, these exercises contribute to the improvement of patient fitness and functional ability, enabling them to achieve positive outcomes even when undertaking home-based rehabilitation or self-management without direct supervision from a healthcare professional [91].

### 3.1. Examples of Exercises

When it comes to the exercises utilized in home-based rehabilitation, there are various approaches, which can be employed. One innovative approach involves incorporating games, which leverage augmented reality (AR) features, such as the color ball game, whack-a-mole [92], fruit ninja [93], or engaging in activities like catching butterflies [94], collecting balloons, or feeding animals [80]. Additionally, these exercises can simulate real-life sports activities, including basketball [95], table tennis, beach volleyball, golf, or archery [82]. The studies referenced in this context highlight the positive impact of these techniques, as patients tend to be motivated while exercising, and the motor outcomes are comparable to those observed in a care-unit setting.

Alternatively, a more traditional approach uses common physiotherapy exercises, which can be divided based on their objective, namely improving the shoulder motion or increasing the shoulder muscles’ strength:

- Range of motion exercises: These exercises aim to improve the flexibility and mobility of the shoulder joint. They include movements such as shoulder flexion (raising the arms forward and upward), shoulder abduction (raising the arms out to the sides), shoulder external rotation (rotating the arm outward), and shoulder internal rotation (rotating the arm inward). These exercises can be performed in standing or sitting positions, and they help increase the range of motion in the shoulder.
- Strengthening exercises: Strengthening exercises focus on building the muscles around the shoulder joint to enhance stability and support. Examples include scapular retraction (squeezing the shoulder blades together), wall push-ups (pushing against a wall at shoulder height), and shoulder rows (using resistance bands or weights to pull the hands toward the body). These exercises help improve the strength and endurance of the shoulder muscles.

These may entail the pre-recording of physiotherapists demonstrating how the exercises should be performed. In a home setting, patients can utilize a system, which provides feedback and guidance during the therapeutic exercises prescribed by a physical therapist while also generating progress reports on the exercises [96]. This combination of guidance and feedback facilitates effective home-based rehabilitation.

### 3.2. What to Monitor during Exercises

Given the complexity of shoulder movements, it becomes necessary to monitor various parameters during the execution of exercises. The commonly monitored parameters include shoulder flexion, shoulder adduction and abduction, and shoulder internal and external rotation [44]. Additionally, exercises often involve side and forward arm raises, mixed presses, mixed circles [42], and they may extend to monitoring other anatomical parts, such as the elbow, wrist, and hand [97,98]. It is essential to monitor parameters beyond simply the spatial position of these body parts to ensure the exercises are performed with the intended quality. Parameters such as angular velocity, interruptions during joint rotation, and variations in muscle strength also warrant close monitoring [81].

To facilitate the monitoring of exercise execution, various systems employ different approaches. Inertial measurement units are commonly used, which provide precise measurements of movement using sensors [99]. Alternatively, cameras can be utilized to capture the three-dimensional movements of the shoulder and elbow joints, employing XYZ Cartesian coordinates [100]. These are only a few examples of the technologies discussed in Section 2, which can be leveraged for exercise monitoring purposes.

### 3.3. Variables to Be Evaluated to Adjust the Exercises

In a more traditional approach to rehabilitation and therapy, healthcare professionals, such as physiotherapists, typically evaluate the patient’s performance during exercises and make necessary adjustments to maximize rehabilitation outcomes [101]. However, in the context of home-based rehabilitation, constant monitoring by healthcare professionals is often impractical. As a result, the monitoring of exercises and evaluation of the patient’s performance are instead facilitated by specialized devices, as discussed in Section 2 of this review.

When assessing the execution of an exercise, various parameters can be measured. These include the superficial electromyography (EMG) of muscles in the shoulder and arm [39], range of movement, movement speed [102], angular acceleration, and angular velocity [103]. Additionally, factors such as exercise duration and completion status [75] can also be considered. By obtaining these parameters, evaluations can be based on performance-based impairment indices, such as the Fugl-Meyer motor scale [104], the Wolf motor arm test [105], the shoulder pain and disability index [106], or the modified motor assessment scale [107]. Having access to these monitoring parameters enables a comprehensive assessment of the patient’s performance, allowing for objective evaluations of their progress in home-based rehabilitation.

### 3.4. User Feedback

When it comes to providing feedback to users, different rehabilitation systems adhere to varying guidelines. Some technologies offer visual correction of exercises, employing mirror rehabilitation therapy for the impaired arm based on superficial EMG and machine-learning results, as exemplified by the ReRebot system [108]. Another approach involves displaying score points, exercise duration, and real-time feedback on the number of repetitions using therapy devices, smartphones, or smartwatches equipped with suitable applications [36]. Feedback can also be conveyed through visual cues, such as different colors on a lamp or monitor, with red indicating poor performance, yellow representing intermediate progress, and green indicating good feedback [81]. In gamified approaches, feedback may be as simple as winning or losing the game, depending on the patient’s performance during exercise execution [82,83].

Providing feedback to patients undergoing home-based shoulder rehabilitation has shown to be highly beneficial. It enhances outcomes by engaging and educating patients, offering support during targeted exercises, and enabling real-time correction of potential mistakes, thereby influencing motor learning and engagement in various ways [109,110,111]. By incorporating effective feedback mechanisms, rehabilitation systems promote patient participation and contribute to improved rehabilitation outcomes.

### 3.5. Progression Criteria

The definition of progression criteria in rehabilitation is dependent on the specific pathology being targeted and the rehabilitation system being employed. In gamification-based approaches, progression is often determined by the scores achieved by patients during the game, reflecting their progress compared to previous scores. Patients strive to reach or surpass their highest previous score, continuously adjusting their performance based on past achievement [81,82,112]. However, in rehabilitation methods, which do not incorporate gamification, individual assessment of monitored parameters is conducted, and the data are then evaluated either by healthcare professionals or machine-learning algorithms to define progression criteria [23,113].

While rehabilitation primarily takes place in a home-based setting using dedicated technologies, evaluation of the rehabilitation progress can still involve therapist involvement. Short training sessions may be conducted where the therapist evaluates the patient’s motor performance [78,99]. This allows the therapist to identify any potential issues in the rehabilitation process and address problems, which the rehabilitation system may not be able to correct on its own.

### 3.6. How Can Progression Be Achieved?

Understanding the progression criteria provides clarity on how to manage the progression of rehabilitation. In gamification approaches, the difficulty of the game and exercises is adjusted based on the patient’s performance. If the exercises no longer present a challenge and there is potential for further improvement in the patient’s physical condition, the game difficulty is increased. Conversely, if the patient’s performance declines, the difficulty is lowered to allow for retraining and the reacquisition of any lost capabilities [82,83]. 

Furthermore, a combination of traditional rehabilitation techniques and home program exercises has shown effectiveness in allowing patients to work beyond their pain thresholds. This approach has demonstrated positive outcomes in rehabilitating shoulder stiffness [114]. In general, most systems and methods consider data extracted from the patient’s most recent performance and adapt subsequent sessions accordingly. By utilizing the patient’s performance data, the rehabilitation program can be customized to meet their specific needs and optimize their progress.

### 3.7. Accuracy, Reliability, Scalability, User Experience, and Potential Ethical Concerns

In the design and development of home-based rehabilitation systems, several factors must be considered to ensure that these systems are effective, user friendly, and ethically responsible. These factors include accuracy, reliability, scalability, user experience, and potential ethical concerns.

Accuracy and reliability are critical factors in the design of home-based rehabilitation systems [115]. These systems must be able to accurately measure and track patient progress, as well as provide reliable feedback and guidance to support the rehabilitation process. To ensure accuracy and reliability, these systems are typically tested and evaluated using standardized testing protocols, and performance data are collected to assess their performance.

Scalability is another important factor in the design of home-based rehabilitation systems [116]. These systems must be able to scale to meet the needs of different user populations, including patients with varying levels of physical ability and rehabilitation goals. To achieve scalability, these systems may incorporate modular components or customizable settings, which allow them to be adapted to the specific needs of individual users.

User experience is also a critical factor in the design of home-based rehabilitation systems [117]. These systems must be easy to use, accessible, and provide a positive overall experience for users. To ensure a positive user experience, feedback from users is often collected and incorporated into future system designs. This feedback may include suggestions for improving ease of use, accessibility, or overall satisfaction with the system.

Finally, potential ethical concerns must also be considered in the design and development of home-based rehabilitation systems [118]. These concerns may include issues such as data privacy and security, informed consent, and the potential for misuse or abuse of these technologies. To address these concerns, measures such as secure data storage and transmission protocols, clear informed consent procedures, and safeguards against misuse or abuse may be incorporated into the design of these systems. These are further detailed in Section 4.1.

In conclusion, the design and development of home-based rehabilitation systems must take into account a range of factors to ensure that these systems are effective, user friendly, and ethically responsible. These factors include accuracy, reliability, scalability, user experience, and potential ethical concerns. By carefully considering these factors in the design process, it is possible to develop home-based rehabilitation systems, which support patients in achieving their rehabilitation goals while also providing a positive user experience.

## 4. Guidelines for Home-Based Rehabilitation Systems

Developing effective home-based rehabilitation systems requires adherence to specific guidelines to ensure optimal user experience, safety, and positive rehabilitation outcomes. Below, we propose a set of guidelines, which should be followed during the development process:

1. User-centric design: Prioritize user needs and preferences when designing the system interface. Create an intuitive and user-friendly interface, which accommodates individuals with varying technological proficiency, ensuring easy navigation and engagement during home-based rehabilitation sessions.
2. Accessibility and inclusivity: Ensure that the system is accessible to a diverse range of users, including those with physical disabilities, visual impairments, and cognitive limitations. Incorporate features such as adjustable font sizes, voice guidance, and alternative input methods to enhance accessibility for all patients.
3. Customization and adaptability: Provide options for customization and adaptation of the system to meet individual patients’ specific needs. Allow for adjustments in difficulty levels, exercise intensity, and duration based on the patient’s progress and capabilities.
4. Real-time feedback and guidance: Incorporate mechanisms to provide real-time feedback and guidance during exercises. Utilize visual cues, auditory signals, or haptic feedback to help patients perform exercises correctly and optimize their rehabilitation progress.
5. Comprehensive progress tracking: Develop a robust system for tracking and recording patients’ progress over time. Monitor metrics such as exercise completion rates, duration, intensity, and improvements in performance. Utilize these data to personalize future exercise plans and set realistic goals for patients.
6. Safety measures and risk mitigation: Prioritize patient safety by integrating safety measures into the system. Use technologies such as motion sensors to detect incorrect movements or provide warnings when patients exceed safe ranges of motion. Implement risk mitigation strategies to prevent injury during exercises.
7. Data privacy and security: Ensure that the system complies with data privacy regulations and incorporates robust security measures to protect patient information. Implement measures to safeguard sensitive data and provide patients with control over their data and their usage.
8. Seamless integration with healthcare professionals: Enable seamless communication and data exchange between the home-based rehabilitation system and healthcare professionals involved in the patients’ care. Facilitate remote monitoring, feedback, and the ability to make informed adjustments to the rehabilitation program based on professional expertise.
9. Artificial intelligence and machine-learning integration: Explore opportunities to integrate artificial intelligence or machine-learning algorithms into the home-based rehabilitation system. Leverage artificial intelligence capabilities to analyze patient data, identify patterns, and provide personalized recommendations for exercise progression, adapting the rehabilitation program based on individual needs and response to treatment.
10. Scalability and compatibility: Design the system to be scalable and compatible with various devices and platforms. Accommodate different technologies and future advancements, ensuring flexibility and long-term viability of the home-based rehabilitation system.

By adhering to these guidelines, researchers and developers can create effective home-based rehabilitation systems, which prioritize user needs, promote engagement, and improve rehabilitation outcomes. These systems contribute to the advancement of rehabilitation practices and enhance the overall well-being of patients undergoing home-based rehabilitation programs.

### 4.1. Ethical Concerns

As technology continues to advance in the field of home-based rehabilitation systems, it is crucial to address the ethical considerations associated with their use. The following ethical concerns should be taken into account when developing and implementing such systems:

1. Privacy and data security: Home-based rehabilitation systems often involve the collection and storage of sensitive personal health information. It is essential to ensure that robust data protection measures are in place to safeguard patient privacy and prevent unauthorized access or misuse of the data.
2. Informed consent: Patients participating in home-based rehabilitation programs should be adequately informed about the purpose, risks, benefits, and potential limitations of the technology being used. Informed consent should be obtained, outlining the scope of data collection, potential risks, and the patient’s right to withdraw from the program at any time.
3. Equity and accessibility: It is important to consider the equitable access and availability of home-based rehabilitation systems to ensure that all individuals, regardless of socioeconomic status or geographical location, have equal opportunities for rehabilitation and benefit from the technology.
4. User autonomy and empowerment: Home-based rehabilitation systems should be designed to empower patients and promote their active involvement in their own care. The technology should support patient autonomy, allowing them to make informed decisions about their treatment and providing them with tools for self-management.
5. Transparent and explainable AI: If machine-learning algorithms are integrated into the rehabilitation systems, there is a need for transparency and explainability. Patients should have a clear understanding of how AI is being used, what factors influence treatment recommendations, and the basis for any decisions made by the system.
6. Clinical oversight and human interaction: While home-based rehabilitation systems offer the advantage of remote monitoring and independent practice, it is essential to maintain a balance between technology-driven interventions and the involvement of healthcare professionals. Regular clinical oversight and human interaction should be incorporated into the system to ensure patient safety, address individual needs, and provide timely support or intervention when required.

Addressing these ethical concerns is paramount to fostering trust, protecting patient rights, and ensuring the responsible and ethical implementation of home-based rehabilitation systems. By considering these ethical considerations, developers and healthcare professionals can work together to create systems, which prioritize patient well-being, privacy, and equitable access to high-quality rehabilitation care.

## 5. Study Limitations

This narrative review has several limitations, which should be acknowledged. Firstly, due to its narrative nature, there is a possibility of bias in the selection and interpretation of the included literature. The absence of a research protocol or predefined hypothesis may have influenced the data selection process. Additionally, this review did not adhere to established guidelines, such as those proposed by PRISMA, and the literature search was conducted in a limited database, which may have restricted the breadth of the included studies.

While this narrative review successfully achieves its main objectives of providing an overview of home-based shoulder rehabilitation technologies and offering insights into prescribed exercises, it should be noted that the amount of information presented is limited. It is important to note that this overview does not include detailed information about the methodologies and measuring parameters used in the existing literature. This is partly because many of the referenced works do not share this information themselves. As such, the analysis presented in this study is limited to a general overview of the technologies and their potential applications, without a critical evaluation of their effectiveness based on empirical data. Future studies could benefit from a more comprehensive analysis, which includes a detailed review of the methodologies and measuring parameters used in the existing literature, where available. In addition, the discussion of each topic is approached theoretically and simplistically, making this review less suitable for researchers seeking in-depth, specific information on any of the technologies discussed.

Future studies should consider addressing these limitations by incorporating a more comprehensive and rigorous approach. This could involve providing more detailed information on each technology or dividing the content into multiple specialized studies, allowing for a more focused exploration of specific aspects.

By recognizing these limitations, we aim to provide transparency and encourage further research to expand upon the findings presented in this narrative review.

## 6. Conclusions

The advancements in software and hardware industries have paved the way for the development of more engaging equipment and services used in shoulder home-based rehabilitation. These innovative rehabilitation systems are the result of a multidisciplinary approach, combining technologies from both the physical and virtual branches discussed in this review. However, it is crucial to acknowledge that the progress in rehabilitation would not be possible without the collaboration between technology and healthcare experts.

To further advance the field of rehabilitation, it is essential to foster knowledge exchange and collaboration between professionals from the technology and healthcare sectors. This can be achieved by establishing partnerships and promoting interdisciplinary research within institutions dedicated to innovation and the advancement of rehabilitation practices.

By bridging the gap between technology and healthcare, we can propel the state of the art in rehabilitation, unlocking new possibilities for effective shoulder rehabilitation in home-based settings. This collaboration holds great potential for enhancing patient outcomes and transforming the field of rehabilitation.

In conclusion, the integration of technological advancements with healthcare expertise offers promising opportunities for improving shoulder home-based rehabilitation. By embracing collaboration and fostering innovation, we can drive the future development of rehabilitation systems and methods, ultimately benefiting patients and advancing the field as a whole.

## Figures and Tables

**Table 1:** Examples of wearable devices for shoulder rehabilitation.

Year	Wearable System Description	Reference
2011	Inexpensive and wearable upper body orthotics system, composed of a soft-orthotic device with an integrated cable actuation system, and an actuator package.	(Kesner et al.) [25]
2012	Soft-orthotic system to monitor and control the patient’s posture.	(Galiana et al.) [22]
2020	Wearable motion sensor device to assist patients in conducting home-based exercises to improve training compliance and the accuracy of exercises.	(Chen et al.) [26]
2019	Portable system without the need for any adjunctive sensor/peripheral interconnection cable and/or marker, which must be worn.	(Condino et al.) [43]
2012	A thin, compliant, lightweight, cost-effective soft-orthotic device with an integrated cable actuation system, which is worn over the upper body, an embedded limb position sensing system, an electric actuator package, and controller.	(Galiana et al.) [22]

**Table 2:** Examples of robots used for shoulder rehabilitation.

Year	Robot Description	Reference
2007	A new adjustable robotic exoskeleton called MEDARM for motor rehabilitation of the shoulder complex.	(Ball et al.) [28]
2016	Low-end robot system for comprehensive shoulder rehabilitation. The hardware features lightweight and low-cost design using only one actuator with five single-DOF shoulder motions achievable.	(Park et al.) [57]
2017	ArmAssist, a simple low-cost robotic system for upper limb motor training, which combines known benefits of repetitive task-oriented training, greater intensity of practice, and less dependence on therapist assistance.	(Tomić et al.) [58]
2019	ANYexo, an upper limb rehabilitation robot, optimized to achieve a large range of movements and mimic the interactions of a therapist.	(Zimmermann et al.) [30]
2021	ASPIRE, a spherical parallel robotic system, which targets the adduction, abduction, flexion, and extension rehabilitation motions of the shoulder joint and the pronation and supination of the forearm.	(Tucan et al.) [29]
2021	MERLIN, an unactuated version of the robot ArmAssist, combined with a telecare platform to rehabilitate stroke patients. This robot uses serious games to train the upper limb in home-based rehabilitation scenarios.	(Rozevink et al.) [59]

**Table 3:** Examples of exoskeletons used for shoulder rehabilitation.

Year	Exoskeleton Description	Reference
2016	A new iteration of a previously developed shoulder exoskeleton, which is small in size, lightweight, requires low power consumption, and contains an adaptive mechanism, which can compensate for the misalignment between exoskeleton and human upper limb and size variation among different subjects.	(Chien et al.) [67]
2017	CLEVERarm, an upper limb rehabilitation exoskeleton with 8 DOF (6 active and 2 passive), supporting the motion of shoulder girdle, glenohumeral joint, elbow, and wrist.	(Soltani-zarrin et al.) [32]
2019	NESM, a passive 4-DOF shoulder-elbow exoskeleton for upper limb rehabilitation and treatment of spasticity.	(Trigili et al.) [34]
2022	A passive shoulder complex exoskeleton with 6 DOF used for self-rehabilitation of post-stroke hemiplegic patients.	(Li et al.) [33]
2022	Hypothesis of a passive bionic portable elbow exoskeleton, based on a human-exoskeleton gravity-balancing coupled model.	(Meng et al.) [35]

**Table 4:** Examples of machine-learning applications used for shoulder rehabilitation.

Year	Algorithm Description	Reference
2015	The iJoint, a telerehabilitation system for patients with frozen shoulder, which uses a machine-learning approach to estimate the angle of rotation using the accelerometer sensors of a smartphone.	(Ongvisatepaiboon et al.) [38]
2018	An open-source motion-based machine-learning application, which uses smartphone accelerometer sensors to measure the shoulder arcs of motion.	(Ramkumar et al.) [36]
2019	Multi-Stream LSTM Dueling (MS-LSTM Dueling), a DL model for predicting trajectories with multi-joint motion of a NTUH-II exoskeleton using inertia measurement units and superficial EMG signals as inputs.	(Ren et al.) [37]
2020	A convolutional neural network algorithm, which processes EMG signals from 12 muscles for the pattern recognition of upper arm motions, including resting, drinking, backward and forward motion, and abduction motion.	(Jiang et al.) [39]
2020	A home-based rehabilitation system, which can detect and identify the rehabilitation exercise of a patient based on a convolution neural network algorithm and smartwatch. The system is also capable of evaluating the clinical outcomes for patients with chronic stroke.	(Chae et al.) [40]

**Table 5:** Examples of virtual and augmented reality systems used for shoulder rehabilitation.

Year	System Description	Reference
2014	RehaBio, a system, which uses patients’ rehabilitation information to track their progress and uses an exercise module to provide exercises based on an AR approach.	(Aung and Al-Jumaily) [44]
2019	A wearable AR application, which implements serious games for shoulder rehabilitation, based on Microsoft HoloLens, with real-time tracking of the user’s hand, with no need for markers/sensors for arm/hand tracking.	(Condino et al.) [43]
2021	An immersive VR-based relaxation program, which provides an array of scenarios, which the patients can select, with the intent to redirect their attention from the pain. Some available scenarios include a “Dream Beach” and “Wild Dolphins”.	(Funao et al.) [46]
2022	An immersive VR system for replicating mobility and strength metrics from successful physical therapy sessions through the application of ML, with patients’ observations as input.	(Powell et al.) [42]
2022	A study comparing the accuracy of a VR system Oculus Quest 2 against an optoelectronic system (Qualisys optical capture system) in the measurement of shoulder translational and rotational displacements. The results show that Oculus Quest 2 is a viable alternative to traditional motion analysis systems in home-based rehabilitation.	(Carnevale et al.) [45]

**Table 6:** Examples of serious games used for shoulder rehabilitation.

Year	Game Description	Reference
2018	ReMoVES, a home-based platform for motion rehabilitation through serious games and biophysical sensors, with motion tracking capabilities through Microsoft Kinect V2 and Leap. The emotional state of the patient is evaluated with heart rate measurements and electrodermal activity monitored by Microsoft Band 2.	(Morando et al.) [48]
2021	This study evaluates the usability of the MERLIN robotic system based on serious games for upper limb rehabilitation in patients with stroke undergoing home-based rehabilitation. The results show that almost every patient found the system useful, safe, and motivating, and all of them achieved moderate clinical improvement in motor function.	(Guillén-Climent et al.) [49]
2022	A study using Oculus Rift and serious games to facilitate the remote identification and classification of human movements for the automatic assessment of motor performance during rehabilitation through the user’s interaction with a VR interface.	(Ventura et al.) [50]

## Data Availability

Not applicable.
